# Initial report of γ-aminobutyric acidergic locomotion regulatory system and its 3-mercaptopropionic acid-sensitivity in metamorphic juvenile of sea urchin, *Hemicentrotus pulcherrimus*

**DOI:** 10.1038/s41598-020-57567-w

**Published:** 2020-01-21

**Authors:** Hideki Katow, Hiromi Yoshida, Masato Kiyomoto

**Affiliations:** 1 Research Center for Marine Biology, Tohoku University, Asamushi, Aomori 039-3501 Japan; 2 Institute of Development, Aging and Cancer, Tohoku University, Sendai, 980-8575 Japan; 3 Marine and Coastal Research Center, Ochanomizu University, Tateyama, Chiba 294-0301 Japan

**Keywords:** Biochemistry, Cell biology, Developmental biology, Immunology, Molecular biology, Neuroscience, Physiology, Structural biology, Zoology

## Abstract

The γ-aminobutyric acid (GABA) signal transmission system (GSTS) contributes to larval swimming through the regulation of ciliary beating. However, whether this system also contributes to the primary podia (PP)-generated motility of juveniles remained unclear. The present study aimed to elucidate the involvement of the GSTS in the motility of metamorphic juveniles (juveniles) (1) by immunohistochemically elucidating the location of molecular constituents of the PP, and (2) by inhibiting the activity of GΑΒΑ decarboxylase (GAD) with 3-mercaptopropionic acid (3-MPA). During metamorphosis, the echinus rudiment protrudes its PP out of the body surface in 8-arm plutei. The PP expresses immunopositive signal (-IS) of GAD, GABA, GABA_A_ receptor and tropomyosin, and is constituted with the GABA-IS negative distal tip and the GABA/GAD-IS gaiter region. The latter radiates distal projections to the disc that contains a GAD-IS cellular network. The juvenile body cavity houses a GABA/βIII-tubulin-IS Penta-radial ring (PrR) that extends branches into each PP and several bridges to the GAD/GABA-IS Penta-radial plate (PrP) on the oral side but does not reach to the gaiter region. 3-MPA reversibly inhibits the juvenile motility and GABA-IS expression in the PrR/PrP complex. This indicates that the complex is the major contributor to the GABAergic motility in juveniles.

## Introduction

The swimming activity of sea urchin plutei is carried out by the body surface ciliary beating and is regulated by various neurotransmitters, including GABA^1^, serotonin^2–4^, and dopamine^5,6^. The locomotive activity of juveniles soon after metamorphosis depends on the primary podia (PP) that are derived from the epineural fold of echinus rudiment (EcR) during the late pluteus stages. The PP are accompanied by the radial nerves that extends from the circumoral nerve ring^7,8^ and, similar to adult tube feet (TF), it uses a solid substrate to walk by a sucking action that is generated at the disc and the contractile activity of the TF muscle^9^.

The TF is comprised of water vessels^8^ and muscle tissue, which is accompanied by the nervous systems (NS) beneath the ciliated epithelium^10^. The NS in the TF is connected to the circumoral nerve ring^8,11^ and its movement is regulated by GABA and acetylcholine^12^. Thus, GABAergic NS involvement in the motile activity throughout sea urchin development appears to be consistently taken over from the ciliary beating in the larval stage^1,13,14^ to creeping movement by the TF of the adult sea urchins. However, how the GABAergic motile organ develops to the adult TF during juvenile development remained unclear. In adult sea urchins, the circumoral synaptotagmin (Syt)-expressing Penta-radial nerve ring appears to control the tube feet movements^15^. Thus, the present study aimed to elucidate the following by means of immunohistochemistry of a GABA-immunopositive signal (-IS), glutamate decarboxylase (GAD)-IS, GABA_A_ Receptor (GABA_A_R)-IS, and tropomyosin (TM)-IS constituents, and through 3-D reconstruction, and pharmaceutical bioassay analysis. They are (1) to elucidate the detailed developmental process of the GABAergic NS of the motile organ from the very late 8-arm pluteus stage PP to the metamorphic juvenile (juvenile) TF, (2) to identify the histological positional relations of molecular constituents, and (3) to determine the immunohistochemical foundation of the juvenile’s motility under interruption of GAD activity by 3-mercaptopropionic acid (3-MPA).

The present study detected extensive GABA-IS blastocoelar cell network connected to the PP by passing through the EcR surface during the early period of metamorphosis in 8-arm pluteus larva. During the further developmental period of PP elongation, GAD-IS, GABA_A_R-IS, TM-IS, and GABA-IS were subsequently detected. They were organized to the concentric pattern by the end of metamorphosis. Such distribution pattern was retained in the PP of juvenile. The GAD-IS ciliary band (CB)-associated strand of the larval arms in 8-arm pluteus larva and the PP of the growing EcR^1^ coexisted until the end of metamorphoses. However, their motility was retained without apparent coordination between the organs. These PP were closely associated with the GABA-IS Penta-radial ring (PrR) and the Penta-radial plexus (PrP) (PrR/PrP complex), which were detected around the mouth of a juvenile. Like the swimming activity of plutei^1,14^, the mobility of juveniles was reversibly interrupted by the presence of 3-MPA, which was accompanied by the decrease of GABA-IS in the PrR/PrP complex.

## Materials and Methods

### Animal preparation

Sea urchins, *H*. *pulcherrimus* (A. Agassiz), were collected in the vicinity of the Research Center for Marine Biology, Tohoku University, Japan or the Marine and Coastal Research Center, Ochanomizu University, Japan. Gametes were obtained by intracoelomic injection of 0.5 M KCl. Eggs were inseminated and incubated in filtered seawater (FSW) on a gyratory shaker or were gently stirred with a propeller in plastic containers in an incubator at 15 °C or 18 °C until the appropriate developmental stages were reached. Larvae were fed with around 10,000 cells/ml *Chaetoceros calcitrans* (Nisshin Marine Tech. Ltd., Yokohama, Japan) from four days after fertilization until the day that is described in the text. Some of the larvae were further incubated and metamorphosis was induced by the method described by Kiyomoto *et al*.^16^.

### Whole-mount immunohistochemistry

The larvae that reached the developmental stages described in the text, and the 1-day post-metamorphosis (1-dpm) to 5-dpm juveniles were fixed in 4% paraformaldehyde (diluted in FSW) for 15 to 20 min at ambient temperature (AmT) and dehydrated in a series of increasing concentration of ethanol starting from 30% (v/v) and stored in 70% ethanol at 4 °C until use. For WMIHC, the samples were hydrated in decreasing concentration of ethanol to 30% and transferred to 0.1 M phosphate buffered saline with 1% (v/v) Tween-20 (PBST; Medicago AB, Uppsala, Sweden).

The samples were blocked with 1% (w/v) bovine serum albumin in PBST for 1 h and exposed to the following primary antibodies (Abs) in PBST for 24 to 48 h at 4 °C (Table 1) in various combinations for multi-stained WMIHC. These Abs were then washed in PBST three times (10 min each) and were visualized with the following secondary Abs that were diluted in PBST. The secondary antibodies used were Alexa Fluor 488-, 568- or 594-tagged goat anti-rabbit or –mouse IgG Abs (Invitrogen, Carlsbad, CA. USA; diluted 1:200 to 1:500), Alexa Fluor 647-tagged goat anti-mouse IgG Ab (Abcam plc. Cambridge, USA; diluted 1:200 to 1:1,000) or Alexa Fluor 488-tagged goat anti-chicken IgY Ab (Abcam; diluted 1:500) and they were incubated with the samples for 2 h at AmT. Most of the samples were counterstained with 1–2 μg/ml 4′, 6-diamidino-2-phenylindole (DAPI) and cleared in Olympus Scaleview-A2 optical clearing solution (Wako Pure Chemical Ind. Ltd. Osaka, Japan) or CUBIC^17^ for a few hours (for 8-arm pluteus larva in early metamorphosis period) or for 3 days (for juveniles) at 4 °C according to the manufacturer’s instructions. They were examined under a Micro-Radiance confocal laser scanning microscope (CLSM; Bio-Rad Microscience, Hemel Hempstead, UK) or a TCS SP8 CLSM (Leica Microsystems, Co. Japan, Tokyo, Japan); otherwise, they were analyzed as described in the text. Images were analyzed with ImageJ (National Institutes of Health, NIH; http://rsbweb.nih.gov/ij/) or Adobe Photoshop CS2 software (ver. 9.02, Adobe Systems Inc., San Jose, CA, USA).

**Table 1:** List of antibodies used in this study.

Antigen	Host	Antibody type	Applied dilution	References
Ap-Synaptotagmin	Mouse	Monoclonal (1E11)	No dilution	Nakajima *et al*.^37^
Hp-GABA_A_R	Mouse	Polyclonal	1:400	Katow *et al*.^1^
Hp-Tropomyosin	Rabbit	Polyclonal	1:200	Ishimoda-Takagi *et al*.^18^
Rat-GAD65/67	Rabbit	Polyclonal	1:500	Millipore, Katow *et al*.^1^
Hp-GAD	Rabbit	Polyclonal	1:500	Katow *et al*.^14^
GABA	Rabbit	Polyclonal	1:50	Millipore
GABA	Mouse	Monoclonal (GB-69)	1:100	Sigma
Human β-III tubulin	Chicken	Polyclonal	1:1,000	Merck (AB9354)

Ap; Asterina pectinifera. Hp; Hemicentrotus pulcherrimus.

### 3-D image construction

To clarify the spatial relationship of the images obtained with CLSM, the optical sections were reconstructed three-dimensionally with the following 3-D visualization and analysis softwares. They are an Avizo software ver.6.1.1 (FEI Visualization Sciences Group, Bordeaux, France), an Amira software (FEI Visualization Sciences Group, Burlington, MA 01803, USA) or an ImageJ 1.45 s (National Institutes of Health, USA).

### Video recording of the metamorphosis process

The late-8-arm pluteus larvae that were in the process of metamorphosis were transferred to FSW in 4 cm diameter plastic dishes, and the behavior of larvae or juveniles at AmT was recorded for 2 to 9 min with an EOS Kiss X3 digital camera (Canon Inc. Tokyo).

### Bioassay for movement of juveniles with or without 3-MPA

**Figure 1:**
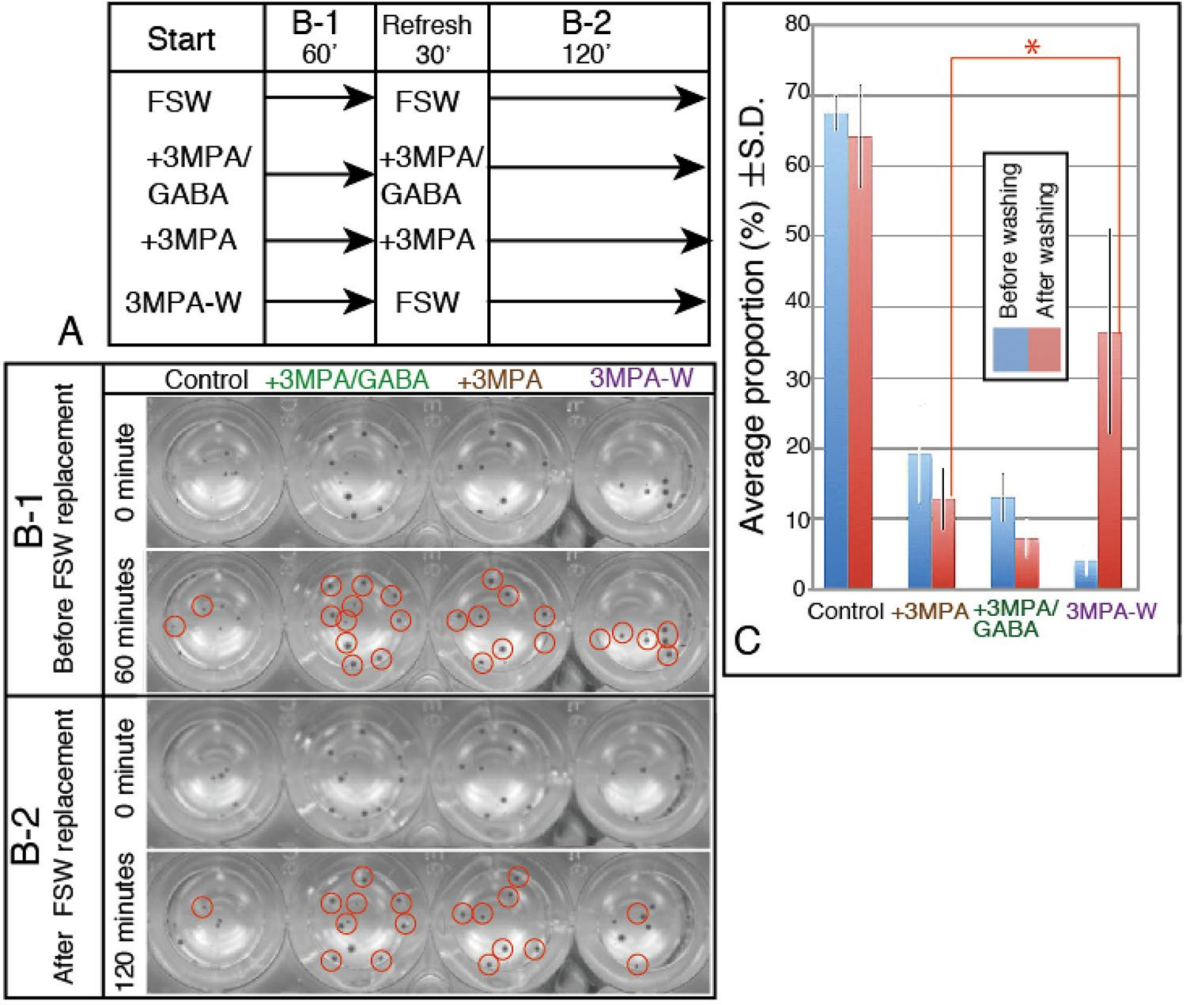
Motility of juveniles with or without 3-MPA. (**A**) Summary of the assay procedures. (**B**-**1**) Representative scanner image of juveniles in 24-well plates that were incubated in normal seawater (Control), 1 μM 3-MPA (+3MPA), a mixture of 1 μM 3-MPA and 10 μM GABA (+3MPA/GABA) or in 1 μM 3-MPA before washing out with plain seawater (3MPA-W). (**B**-**2**) Representative scanner image of juveniles replaced with fresh plain seawater (Control), fresh 3-MPA (+3-MPA), 3-MPA or GABA (+3-MPA/GABA) or plain seawater (3-MPA-W). Red circles, juveniles that unchanged their location from the initial position during the period indicated on the left side boxes. (**C**) Statistical analysis of the proportion of juveniles that changed their position (creeped) during the incubation period. Asterisk; Subjected pair of assay results with significant difference (P = 0.0013). Vertical bars; s.d.

A total of 171 3-dpm juveniles were separated into the following three groups: (1) plain FSW (39 juveniles; Control), (2) 1 μM 3-MPA in FSW (88 juveniles; 3-MPA), and (3) 1 μM 3-MPA + 100 μM GABA in FSW (44 juveniles; 3-MPA+GABA). Prior to the first assay period, each group was further split into five wells of the 24-well plate (Coster, Corning, NY14831, USA). Each well contained seven to eleven juveniles. The juveniles were incubated for 1 h at AmT. Then, the control group was replaced with fresh plain FSW, and the 3-MPA group was split into the following two groups: one group was treated with replaced fresh 1 μM of 3-MPA, and the other was washed three times (5 min each) with fresh FSW and was further incubated in FSW; the 3-MPA+GABA group was replaced with a fresh 3-MPA+GABA. They were further incubated for 2 h with recording as stated above for the second assay period (Fig. 1A), while their respective positions in the wells at every 15 min by an image scanner GT-8200 UF (Seiko Epson Co., Suwa, Japan) at 300 dpi, 768 × 1024 pixel, 16 bit gray. The assay was repeated three times. The motile behavior of juveniles was examined with an animation made by ImageJ1.47v (National Institutes of Health) with the aid of Java 1.6.0_65 (32-bit) (Oracle Co., Redwood Shores, CA 94065. USA).

An initial analysis of the motile behavior was conducted based on the proportion of juveniles that changed their position in a well from the initial settled place for 1 h for “before washing” and, from the initial place after FSW exchange for 2 h for “after washing” after 30 min rest. The statistical significance of average proportions of juveniles that changed from initial positions between the two groups (“before FSW replacement” and “after FSW replacement”) were analyzed using an unpaired *t*-test using the public-domain software QuickCalcs GraphPad (http://www.graphpad.com/quickcalcs/ttest1.cfm). Two-tailed P values of less than 0.05 were regarded as statistically significant differences.

A statistical analysis of the number of juveniles that expressed the GABA-IS PrR/PrP complex was conducted using samples that were fixed soon after the 3-MPA assay test, as described above. A total of 22 juveniles (7 for the control, 9 for 3-MPA, 3 for 3-MPA+GABA and 3 for washed after 3-MPA) were triple stained with mouse anti-GABA monoclonal antibody (mAb) (Sigma), anti-TM rabbit antibody^18^ and DAPI.

## Results

### Primary podia formation from the late 8-arm pluteus larva stage to the end of metamorphosis

**Figure 2:**
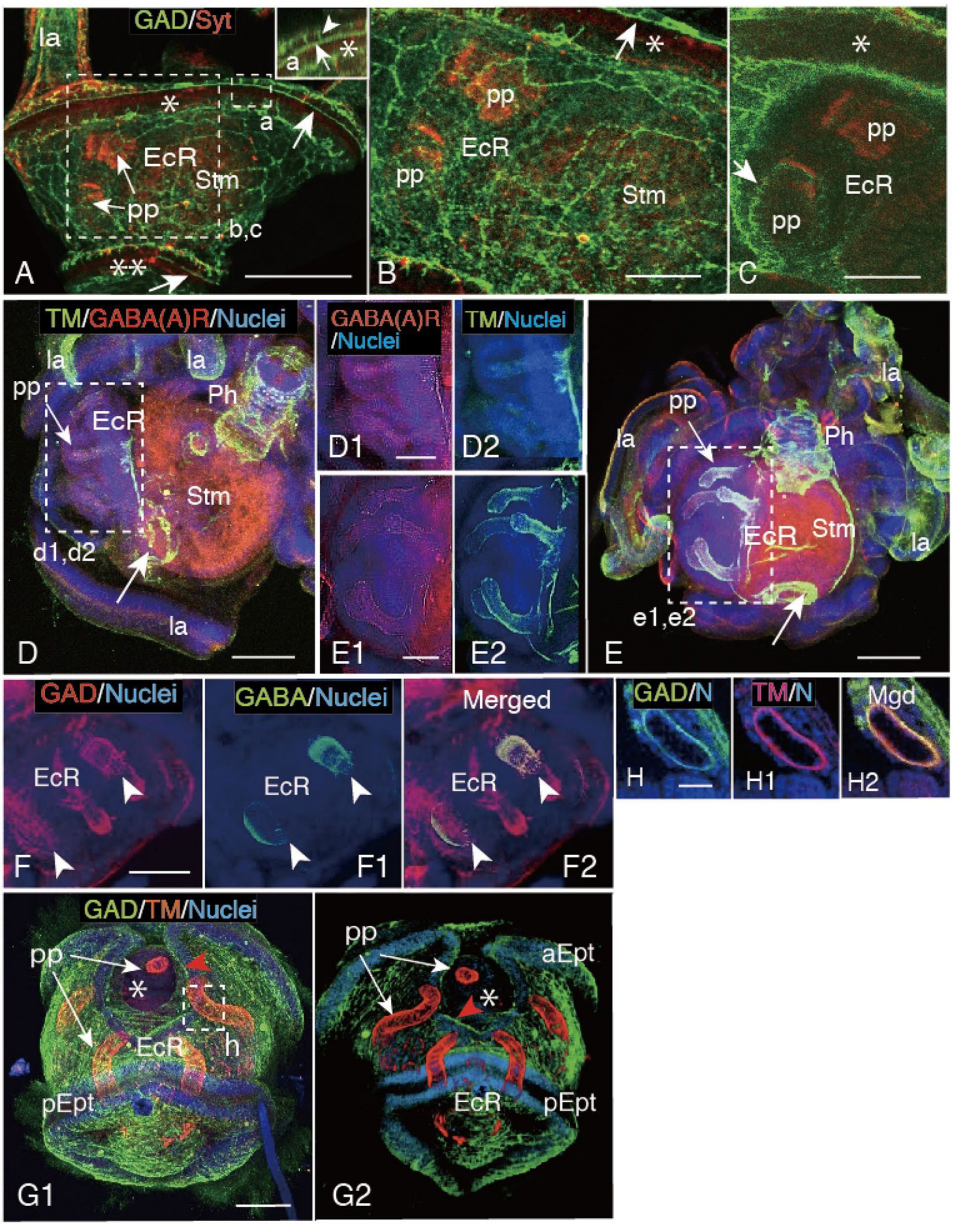
Immunohistochemistry of primary podia (PP) development in the echinus rudiment (EcR) of 8-arm plutei. (**A**) Left-side trunk. Inset (a), higher magnification of a box (a). Arrow; ciliary band-associated strands. Arrow head; connection between the anterior epaulette (asterisk) and the GAD-immunopositive (GAD-IS) blastocoelar network. Dual asterisks; posterior epaulette. (**B**) Higher magnification shown by a box (b,c) in (**A**). Arrow; ciliary band-associated strand. Asterisk; anterior epaulette. (**C**) Optical cross-section of EcR shown by a box (b,c) in (**A**). Arrow; GAD-IS PP surface. Asterisk; anterior epaulette. (**D**,**E**) Older plutei than (**A**). Arrow; anus muscle ring. (**D**) PP-encased EcR. (**D1**) Higher magnification of a box (d1,d2) in (**D**) shows GABA_A_R-IS PP. (**D2**) The same area as (**D1**) shows faint tropomyosin (TM)-IS PP at a similar region to GABA_A_R-IS sites in (**D1**). (**E**) Slightly older pluteus than (**D**). (**E1**) Higher magnification of a box (e1,e2) in (**E**) shows faint GABA_A_R-IS PP. (**E2**) Same area as (**E1**) shows intensified TM-IS PP. (**F**) Further developed GAD-IS PP than (**E**). (**F1**) Asynchronous GABA-IS expression among PP. (**F2**) Merged image between (**F**) and (**F1**). Both ISs are missing at the tip of PP (arrowheads). (**G1**,**G2**) Three-D image of trunk. aEpt; anterior epaulette. pEpt; posterior epaulette. Asterisk; larval opening. (**G1**) Outer surface view by ImageJ. Red arrowheads; ciliary band-associated strand. (**G2**) Inside out image of (**G1**) by Avizo. (**H**-**H2**) Optical cross-section of a PP shown by box (h) in (**G1**). Red arrowhead; periphery of the opening. (**H**) Optical cross-sections of GAD-IS image. (**H1**) TM-IS image. (**H2**) Merged image between (**H**) and (**H1**). la; larval arm. N; nuclei, Ph; pharynx. Stm; stomach. TM; tropomyosin. Scale bars = 100 μm (**A**,**D**,**E**,**F**), 50 μm (**B**,**C**,**D1**,**E1**), 75 μm (**G1**), 20 μm (**H**).

Consistent with our previous report^7^, PP were detected in the larva as Syt-IS small protrusions in EcR (Fig. 2A, pp). GAD-IS network constituted the basal surface of the blastocoel (the Blastocoel basal GAD-IS-network) and connected to the ciliary band-associated strand in some places [Fig. 2A, arrows, inset (a), arrowhead; 2B, arrow]. According to the optical cross-sections, some of the basal GAD-IS network surrounded EcR (Fig. 2B) and reached to the surface of PP (Fig. 2C, arrow).

In slightly older plutei than that mentioned above, the EcR bulged slightly toward the left side of the larval body surface, and TM-IS and GABA_A_R-IS were faintly detected in all five PP (Fig. 2D,D1,D2). In those whose EcR was more enlarged, GABA_A_R-IS remained weak (Fig. 2E,E1), while the PP presented more intensive TM-IS (Fig. 2E; pp, E2). In further developed plutei, the GAD-IS PP (Fig. 2F) asynchronously expressed GABA-IS among some of the PP (Fig. 2F1,F2). Both ISs were weak at the distal tip of the PP (Fig. 2F-F2; arrowheads). In the plutei that close to the end of the metamorphosis, the TM-IS PP (Fig. 2G1,G2) extended further, and a body surface opening was seen immediately above the EcR on the anterior side of the anterior epaulette (Fig. 2G1,G2, asterisks). According to optical cross-sections of a PP [box (h) in Fig. 2G1], it also was GAD-IS (Fig. 2H-H2).

**Figure 3:**
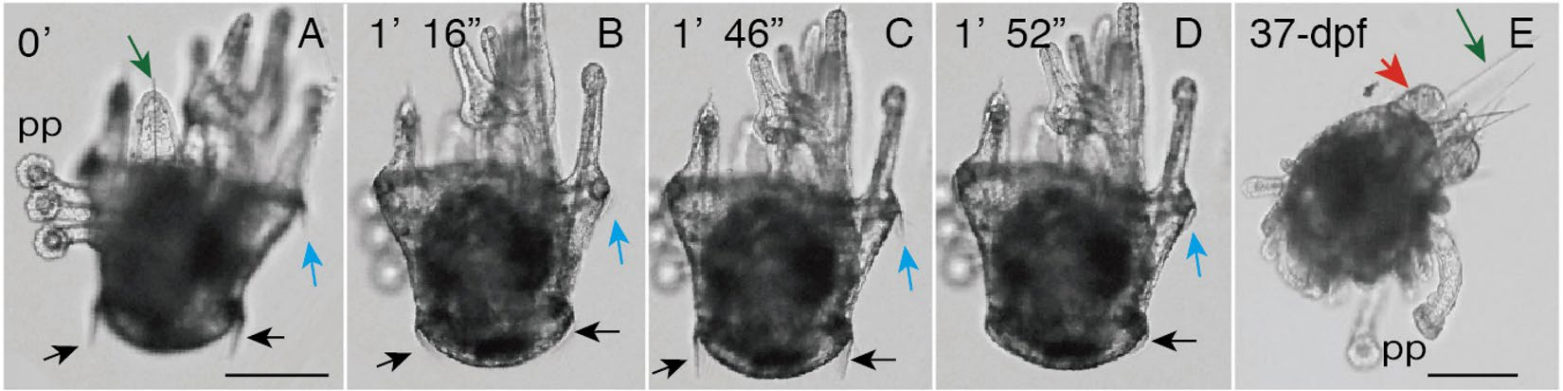
Snapshots of video movie of simultaneous activity of the primary podia (PP) and the ciliary beating of epaulets of 36-day post fertilization larva (**A**–**D**) during very late metamorphosis and 37-dpf juvenile (**E**). (**A**–**D**) Synchronized up-and-down movements of cilia of anterior (blue arrow) and posterior epaulets (black arrows). (**E**) Larval arms retreated (red arrow) by leaving spicules (green arrows). Numbers at the upper left corner; the time started from (**A**–**D**). Scale bars = 150 μm.

The video images of the metamorphosis of 36-dpf plutei indicated that all five PP were extended from the larval body surface and utilized for the creeping movement that resulted in shifting the visual focusing level of PP during the observation (Fig. 3A–D). However, occasionally they detached from the substrate and swam actively with the ciliary beating at both anterior and posterior epaulettes. The occurrence of the creeping movement by PP and swimming by the ciliary beating at the epaulets occurred with mixed combinations, which suggested little or no coordination systems presented between these two motile activities during metamorphosis. The larval arms were retained but were considerably shortened accompanied by apical exposure of internal arm spicules (Fig. 3A, green arrow). By the 37-dpf stage, the larval arms were further absorbed (Fig. 3E, red arrow), and a trace of the arms was left with long spicules (Fig. 3E, green arrow). They did not show the ciliary beating activity on their body surface.

### GABA/GAD-IS expression pattern in PP of early juveniles

**Figure 4:**
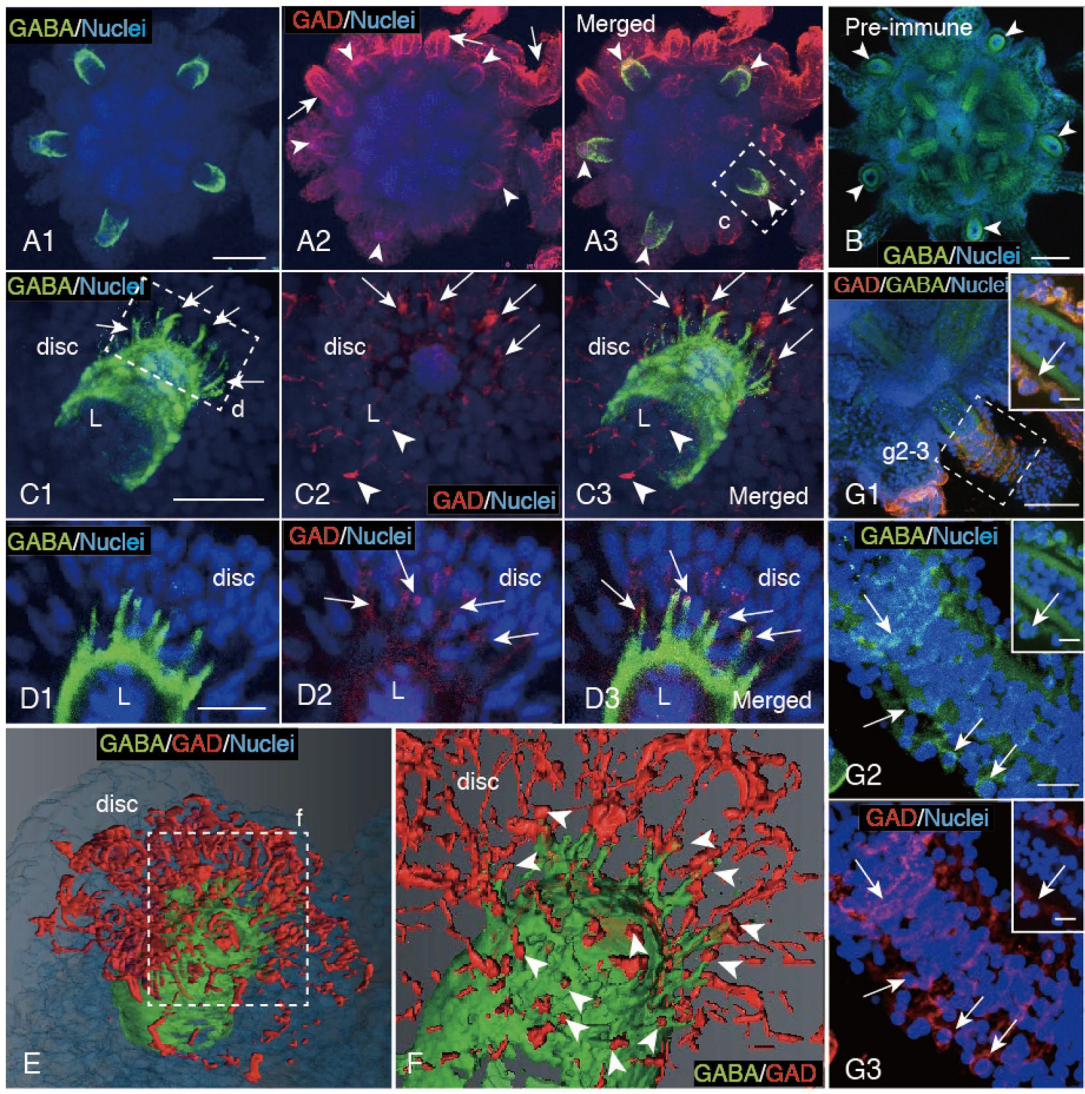
GABA/GAD-IS patterns in the primary podia (PP) of a 3-day post metamorphosis juvenile. (**A1**) GABA-IS PP. (**A2**) GAD-IS on PP (arrowheads) and the larval body surface (arrows). (**A3**) Merged image between (**A1**) and (**A2**). (**B**) Negative control with rabbit pre-immune serum did not detect GAD-IS at the PP (arrowheads) or any part of the body surface. (**C1**–**C3**) Higher magnification of a PP shown by a box (c) in (**A3**). (**C1**) GABA-IS PP is accompanied by a packed spiral pattern and the distal projections (arrows). (**C2**) GAD-IS dots around the distal tip of PP (arrows). (**C3**) Merged image between (**C1**) and (**C2**). (**D1**–**D3**) Higher magnification of a longitudinal optical cross-section of a PP shown by a box (d) in (**C1**). (**D1**) GABA-IS distal projections radiate into the disc. (**D2**) GAD-IS spots in the disc (arrows). (**D3**) Merged image between (**D1**) and (**D2**) shows that the tip of GABA-IS distal projections of PP are double stained with GAD-IS spots (arrows). (**E**) Three-D image of distal tip of a PP by Amira shows close localization of GAD-IS fibers with the GABA-IS distal tip of PP in the disc. (**F**) Higher magnification of the distal tip of PP shown by a box (f) in (**E**) without nuclei staining. The distal tips of GABA-IS projections co-localize with GAD-IS fibers (arrowheads). (**G1**–**G3**) Relaxed PP. Inset; higher magnification of a longitudinal optical cross-section shown by a box (g2-3). Arrows; cell body. (**G1**) GABA/GAD-IS spiral rings around the proximal end of PP. (**G2**,**G3**) Higher magnification of a box (g2-3) in (**G1**). (**G2**) GABA-IS spiral rings. (**G3**) GAD-IS spiral rings. L; luminal epithelium of PP. Scale bars = 75 μm (**A1**,**B**), 25 μm (**C1**), 15 μm (**D1**), 50 μm (**G1**), 20 μm (**G2**), 10 μm (Insets).

A GABA-IS pattern in the PP of EcR in the late 8-arm pluteus larva stage (Fig. 2F1) was inherited by juveniles (Fig. 4A1). GAD-IS on the EcR surface (Fig. 2G) was also inherited by the juvenile body surface (Fig. 4A2,A3,B for negative control). In the PP, GABA-IS was detected accompanied by a spiral ring pattern around the central lumen that radiated distal spiny projections at the distal end into the disc region (Fig. 4C1, arrows). GAD-IS was also detected in the luminal wall of the PP (Fig. 4A2,A3,C2,C3 arrowheads) and in the disc associated with spots around the tips of distal spiny projections (Fig. 4C2, arrows). A merged image localized GAD-IS spots around the tips of GABA-IS distal spiny projections (Fig. 4C3, arrows). The higher magnification image of the GABA-IS distal spiny projection region (Fig. 4D1) depicted apparent partial overlapping with the GAD-IS spots (Fig. 4D2, arrows; D3, arrows). 3D images of the distal spiny projection that were reconstructed based on the above CLSM images indicated that GAD-IS spots actually constituted a network in the disc region around the distal end of PP (Fig. 4E). Each tip of the GABA-IS distal spiny projection was enveloped by the GAD-IS network (Fig. 4F, arrowheads). This could be the structural basis of interaction between the GAD/GABA-IS areas at the distal spiny projection of the PP.

In the relaxed PP, GABA/GAD-IS spiral rings were eminently detected (Fig. 4G1–G3). Both GABA-IS (Fig. 4G1, arrows) and GAD-IS (Fig. 4G3, arrows) were apparently colocalized at the spiral rings, which suggested a functional form of the GAD-GABA-TM complex for the creeping movement of the juvenile by the PP.

### GABA_A_R-GABA-TM-IS localization in the PP of older juvenile

**Figure 5:**
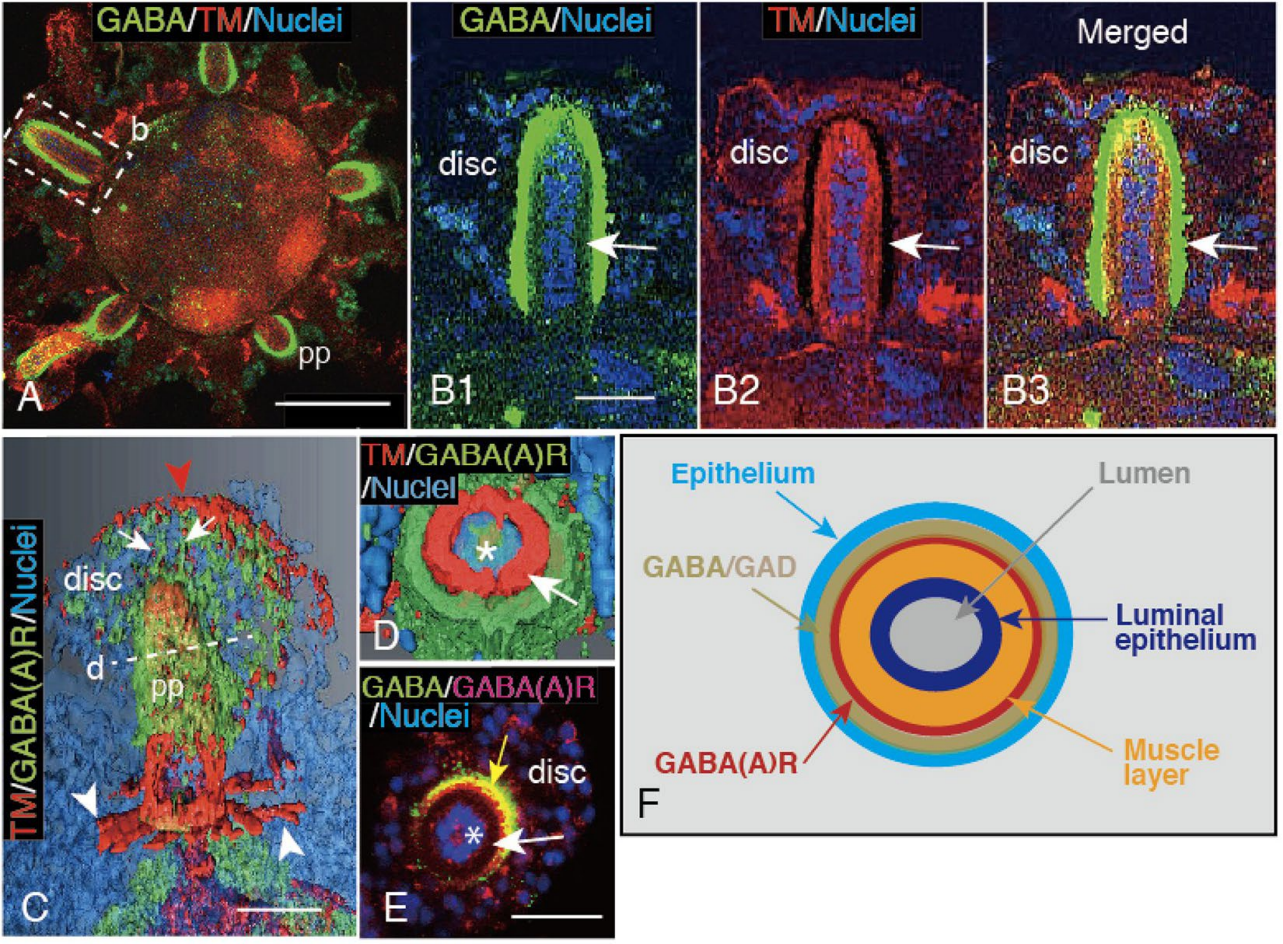
Layered distribution of the GABA-IS area and the tropomyosin (TM)-IS muscle of PP of 5-day post metamorphosis juvenile. (**A**) Oral side view of the juvenile. (**B1**–**B3**) Higher magnification image of a PP indicated by a box (b) in (**A**). (**B1**) GABA-IS outermost layer on the luminal epithelium with a weak IS gap in between (arrow). (**B2**) An immuno-negative dark layer (arrow) surrounds the TM-IS layer on the outer surface of the luminal epithelium. (**B3**) Merged image between (**B1**) and (**B2**) positions the central luminal epithelium, TM-IS layer, and the outermost GABA-IS layer (arrow). (**C**) Three-D image of a PP by Amira. Arrows; GABA_A_R-IS projections. White arrowheads; circumoral TM-IS muscle ring. Red arrowhead; TM-IS peripheral area of the distal disc (disc). (**D**) Three-D reconstructed optical cross-section of a PP by Amira at the gaiter region indicated by a dotted line (d) in (**C**) shows the concentric distribution of TM-IS muscle layer and the GABA_A_R-IS layer on the luminal epithelium (asterisk). (**E**) Concentric distribution of GABA_A_R layer and GABA-IS layer on the luminal epithelium (asterisk) via an immuno-negative space (arrow) shown by a stacked confocal laser scanning micrograph of an optical cross-section of a PP. Two IS areas are partially overlapped (yellow arrow). (**F**) Schematic summary of a PP cross-section shows the concentric distribution of the GAD/GABA-IS layer (light brown), GABA_A_R-IS layer (red) and TM-IS muscle layer (orange). Dark-blue; luminal epithelium. Gray; lumen of PP. Light blue; the outer most epithelium of PP. Scale bars = 100 μm (**A**), 30 μm (**B1**), 25 μm (**C**,**E**).

In the PP of EcR, an expression of GABA-IS (Fig. 2F-F2) was not chronologically well synchronized with TM-IS and GABA_A_R-IS (Fig. 2D1,D2,E1,E2,F1,F2), despite the similar GABA-IS, TM-IS and GABA_A_R-IS pattern (Fig. 5) after metamorphosis. In the 5-dpm juveniles GABA-IS was detected on the TM-IS layer of the PP (Fig. 5A). A more detailed examination of the PP using higher magnification images detected a gap between the GABA-IS layer and the luminal epithelium (Fig. 5B1, arrow). The same PP area indicated that TM-IS layer immediately on the luminal epithelium with a clear IS-negative space between the surrounding area (Fig. 5B2, arrow). The merged image between GABA-IS and TM-IS images positioned the GABA-IS precisely at the previous gap over the TM-IS area (Fig. 5B3, arrow), which indicated that both IS-areas were closely associated with the luminal epithelium of the PP.

To examine the possible involvement of GABA_A_R in close association with the GABA-IS area with the TM-IS layer, the PP of the 5-dpm larvae was double-stained with these antibodies, and the merged image was 3-D reconstructed (Fig. 5C,D). The TM-IS and GABA_A_R-IS area were localized together at PP. The resultant GABA_A_R-IS area was also accompanied to the disc at the distal end (Fig. 5C, arrows), which resembles the structure detected as GABA-IS distal spiny projection of the PP (Fig. 4C1,C3). A 3-D image of an optical cross-section of PP located GABA_A_R-IS layer immediately on the outer surface of the TM-IS layer on the luminal epithelium (Fig. 5D, arrow). A CLSM image of the PP’s optical cross-section that was double-stained for GABA-IS and GABA_A_R-IS detected both ISs negative space immediately on the luminal epithelium (Fig. 5E, white arrow), which implicated that the muscle layer resided there. Thus, the spatial location of PP-constituting proteins indicated a concentric distribution as summarized by Fig. 5F.

### The GABAergic PrR/PrP complex in the juvenile body cavity

A GABA-IS Penta-radial structure in the middle of the body cavity of the juvenile radiated five branches toward each of PP (Fig. 4B, arrowheads). Thus, the next question about the creeping movement regulation was to specify the molecular constituents of that potentially involved in the signal transmission to PP.

**Figure 6:**
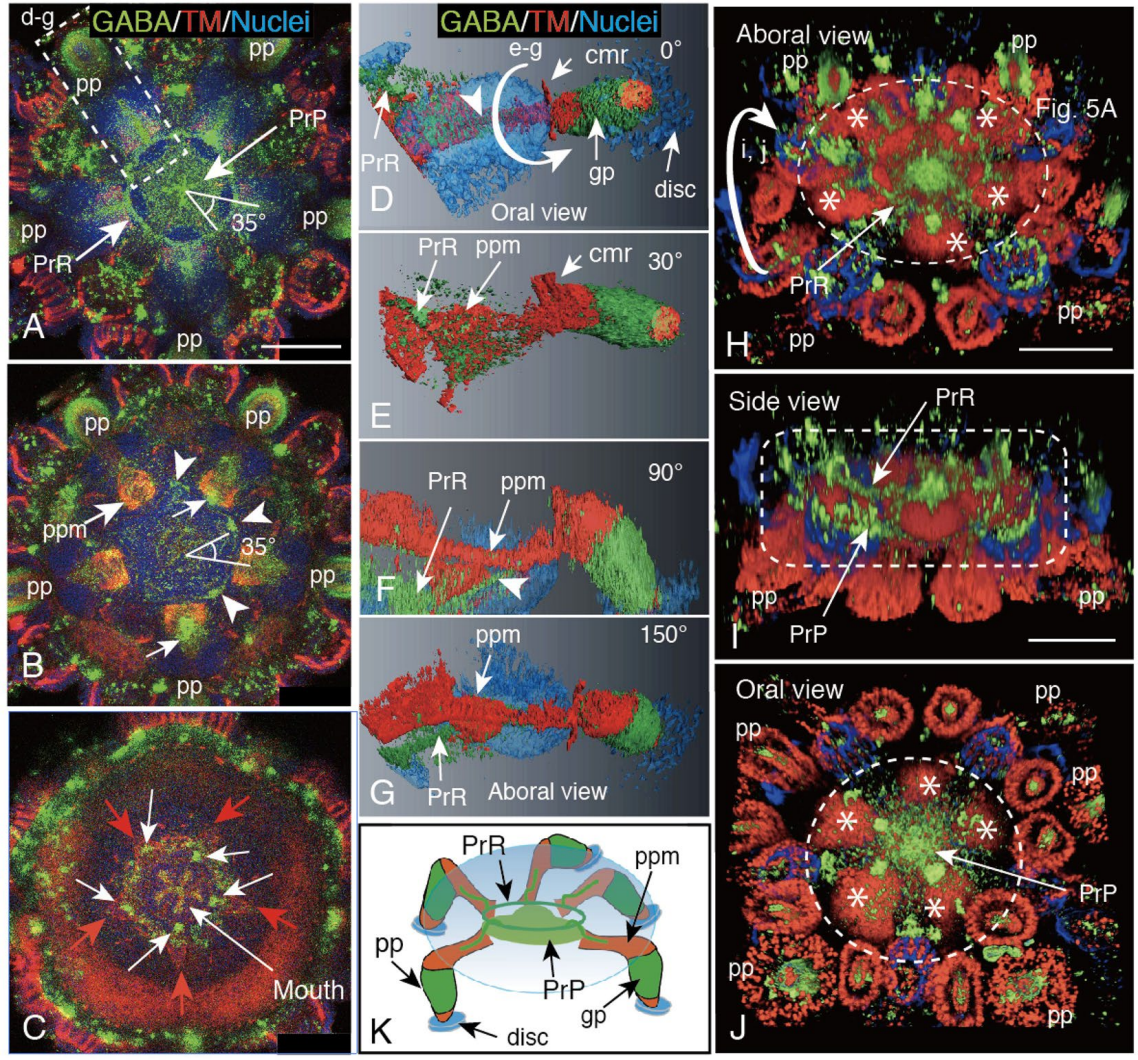
GABAergic PrR/PrP complex in the body cavity of 3-day post-metamorphosis juvenile. (**A**–**C**) Stacked optical cross-sections made perpendicularly to the oral-aboral axis at three levels. Triangle with 35°; the intersection between PrR and PrP at 35°. (**A**) Thirty-micrometer thick image at the level between 78 μm–108 μm from the oral surface (ventral side) of the body shows GABA-IS PrR/PrP complex. (**B**) Six-micrometer thick image at 72 μm–78 μm level shows pentagonal alignment of proximal tropomyosin (TM)-IS muscle regions of PP. Arrows; GABA-IS proximal area of PP. Arrowheads; outer periphery of GABA-IS PrP. (**C**) Six-micrometer thick image at 30 μm–36 μm level shows pentagonal TM-IS triangular shapes (red arrows) that accompanied GABA-IS proximal dots (arrows). (**D**–**G**) Three-D image of a PP by Amira showed by a box (d–g) in (**A**) with various angles (numbers shown at the upper right corner). Curved arrow; rotation direction shown by a curved-arrow (**e**–**g**). Arrowhead; a branch of Penta-radial projection of PrR. cmr; circumoral muscle ring. disc; disc of PP. gp; gaiter of PP. (**H**–**J**) 3-D images of (**A**,**B**) rotated as shown by a curved arrow (i,j). Asterisk; muscle of PP. Circle or rectangle of dotted-line; PrR/PrP complex area. (**H**) Aboral view (dorsal view). GABA-IS PrR is embedded in PP muscle. (**I**) Side view after rotation shows PrR on the aboral side and PrP on the oral side (ventral side). (**J**) Oral view shows PrP resided in the middle of Penta-radially arranged PP muscles. (**K**) Schematic summary showing dorso-ventrally aligned PrR-PrP complex at the center of body cavity (oval of pale-blue gradation). PP or pp; primary podium. PrP; Penta-radial plate. ppm; primary podium muscle. PrR; Penta-radial ring. Scale bars = 75 μm (**A**,**H**).

A series of optical cross-sections of a juvenile localized a Penta-radial GABA-IS ring (Fig. 6A, PrR) and a Penta-radial plexus at the center of the ring (Fig. 6A, PrP) on the aboral side which radiated GABA-IS branches into each of the five PP in 83.4 ± 16.7% of the 8 juveniles examined. Five corners of the central PrP (Fig. 6B, arrowheads) and of the peripheral PrR intersected at an angle of approximately 35° (Fig. 6A,B, triangle), which suggested that apparent ventral fragmental GABA-IS ring may not be a part of the dorsal PrR. The next optical cross-section on the further oral side detected five TM-IS clumps at the proximal end of the PP (Fig. 6B, ppm) with GABA-IS dots on the proximal side (Fig. 6B, arrows). They surrounded a central GABA-IS fragmental ring of the PrP (Fig. 6B, arrowheads). In the very oral end of the optical cross-section, five TM-IS triangles (Fig. 6C, red arrows) and five small GABA-IS dots (Fig. 6C, white arrows) were detected around the mouth.

To elucidate the spatial relationship between the PP constituents and the surrounding tissues, an animation of the 3-D image of a PP of the same CLSM data as in Fig. 6A (box “d-g” region) was reconstructed (Fig. 6D–G). According to the 3-D animation, TM-IS constituted a central column of the PP (Fig. 6E–G, ppm; Supplementary Video S1) all the way to the distal tip, which reached to the disc (Fig. 6D, disc). TM-IS area also was connected to the muscular ring on the oral side (Fig. 6C, red arrows). The GABA-IS area encircled to form a gaiter region on the distal half of the PP (PP gaiter; Fig. 6D–G, gp) but was disconnected from the proximal GABA-IS ring via proximal TF muscle region (Supplementary Video S1). The latter muscle region retained the connection to PrR (Fig. 6D–G, PrR).

The aboral view of the ring was shown to be embedded in TM-IS muscles of PP that orally extended (Fig. 6H, pp). Its side view indicated the GABA-IS aboral layer (Fig. 6I, PrR) and the oral layer (Fig. 6I, PrP). The oral side view revealed five discs of the PP and a GABA-IS plate (Fig. 6J, PrP). Thus, two GABA-IS structures were detected there, and their spatial relationship with PP was shown by a schematic summary (Fig. 6K).

**Figure 7:**
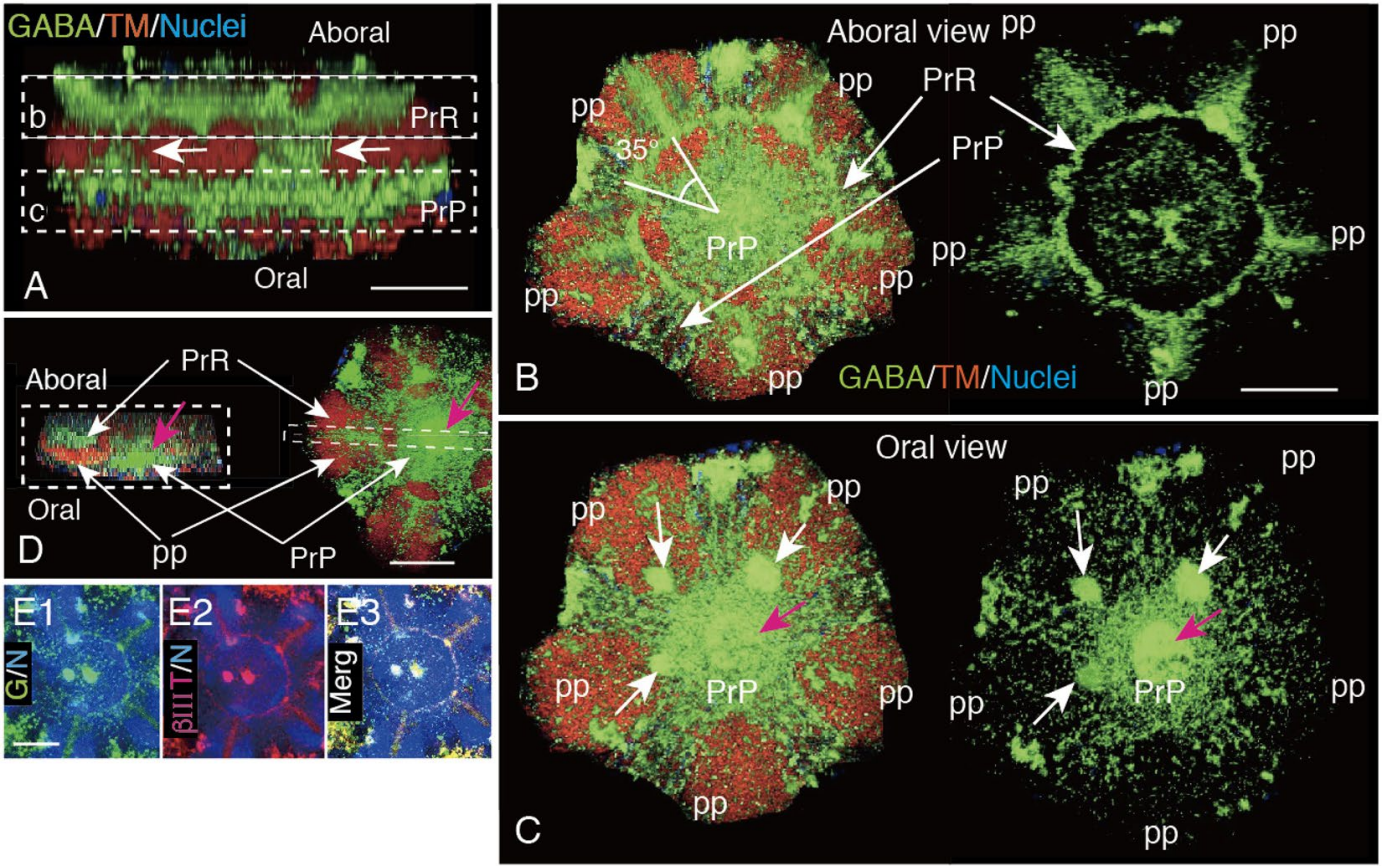
Immunohistochemical property of the GABA-IS Pent radial-ring (PrR)/Penta-radial plate (PrP) complex. (**A**–**D**) Extracted image of a PrR/PrP complex shown by a box and circles in Fig. 6H–J. (**A**) Side view shows connections between PrR and PrP by several GABA-IS cables (arrows). (**B**) Optical horizontal cross-section shown by a box (b) in (**A**). The PrR was localized on the tropomyosin (TM)-IS PP muscle (left) and its five arms diagonally crossed PrP between PP. The PrR was clearly visualized after digital deletion of the TM-IS area (right). Triangle with 35°; intersection between PrR and PrP at 35°. (**C**) Optical horizontal cross-section shown by a box (c) in (**A**). The PrP alone is visualized between the TM-IS PP (left). The PrP is eminently seen after digital removing the TM-IS area (right). (**D**) A perpendicular optical cross-section of the PrR/PrP complex (left) shown by a dotted box on the right image of PrR on the aboral side and PrP on the oral side. (**E1**–**E3**) Colocalization of GABA-IS and βIII-tubulin-IS of 3-dpm juvenile PrR. Fluorescein colors were switched using ImageJ to retain the consistency with the GABA-IS of the others in this figure plate. (**E1**) GABA-IS/Nuclei (G/N). (**E2**) βIII-tubulin-IS/Nuclei (βIII/N). (**E3**) Merged image between (**E1**) and (**E2**). Red arrows: central conical rise of PrP. Scale bars = 30 μm (**A**), 50 μm (**E1**), 100 μm (**B**,**D**).

To ensure the positional relationship of these GABA-IS Penta-radial structures, a 3-D animation was reconstructed using above same CLSM data as shown in Fig. 6A–C. The side view revealed several bridges between the PrR and the PrP (Fig. 7A, arrows). Then, the PrR/PrP complex was digitally split horizontally at the middle of two GABA-IS layers [Fig. 7A, boxes (b) and (c)]. The aboral slice [Fig. 7A, box (b)] detected PrR on the aboral side of TM-IS PP and the PrP between TM-IS PP (Supplementary Video S2A). The PrP intersected the PrR at an angle of approximately 35° (Fig. 7B). In the oral slice [Fig. 7A, box (c)] the PrP was detected only between the TM-IS PP muscles (Fig. 7C, Supplementary Video S2B). The oral-aboral positioning was confirmed also by using an optical cross-section made at the middle of a PrP, and an aboral PrR and an oral PrP (Fig. 7D). This cross-section also detected a conical dome that raised toward the aboral side at the center of the PrP (Fig. 7D, red arrow). Thus, the PrR and the PrP constituted a structurally united complex.

In addition to the GABA-IS PrR, the 3-dpm juvenile exhibited βIII-tubulin-IS (Fig. 7E1–E3), which is a neuron-specific microtubule element^19^.

### Juvenile mobility inhibition by 3-MPA

The aforementioned data suggest that the GABAergic composition of PP reveals neurotransmitter involvement in the PP-dependent movement of the juvenile. To ensure that idea physiologically, the movement of 3-dpm juveniles was assayed under the presence or absence of 3-MPA. The assay procedure was constituted with two periods, as described in the Materials and Methods, and summarized by Fig. 1A. After the initial 60 min of the assay period, the motility of the three groups were severely inhibited, except for the control juveniles (Fig. 1B-1; red circles are juveniles that did not move), which indicated that the inhibitory effect of 3-MPA was not rescued by the exogenous GABA. After the second 120 min of the assay period, while the control juveniles crept actively, those that replaced with fresh seawater after the initial 3-MPA treatment (3-MPA-W) restored the active creeping, indicated that the 3-MPA effect was invalidated by the washing. However, the juveniles that constantly incubated with 3-MPA and those incubated in a mixture of 3-MPA and 1 μM GABA (3-MPA+GABA) retained inactive motility, indicating exogenous GABA did not restore the 3-MPA-induced suppression. (Fig. 1B-2; red circles are those that did not move). Similarly, the average proportion of juveniles that moved throughout the assay period was similar before and after the washing in the control at 67.51 ± 2.46 (Fig. 1C, “before”; blue columns) and 64.14 ± 7.3 (Fig. 1C, “after”; red columns), 19.22 ± 6.96 (before) and 12.83 ± 4.44 (after) in 3-MPA treated (before) that was incubated with fresh 3-MPA (after). Addition of GABA to the 3-MPA medium did not significantly restore the movement from 13.05 ± 3.3 (before) to 7.2 ± 2.62 (after). However, washing out the inhibitor with normal seawater significantly restored the motility (two-tailed P value = 0.0013) from the initial 4.13 ± 2.16 (before) to 36.57 ± 14.6 (after). Thus, the creeping movement was found to be dependent on the GABAergic signal transmission system.

### 3-MPA-independent expression of GABA-IS at PP and -dependent expression at PrR/PrP complex

**Figure 8:**
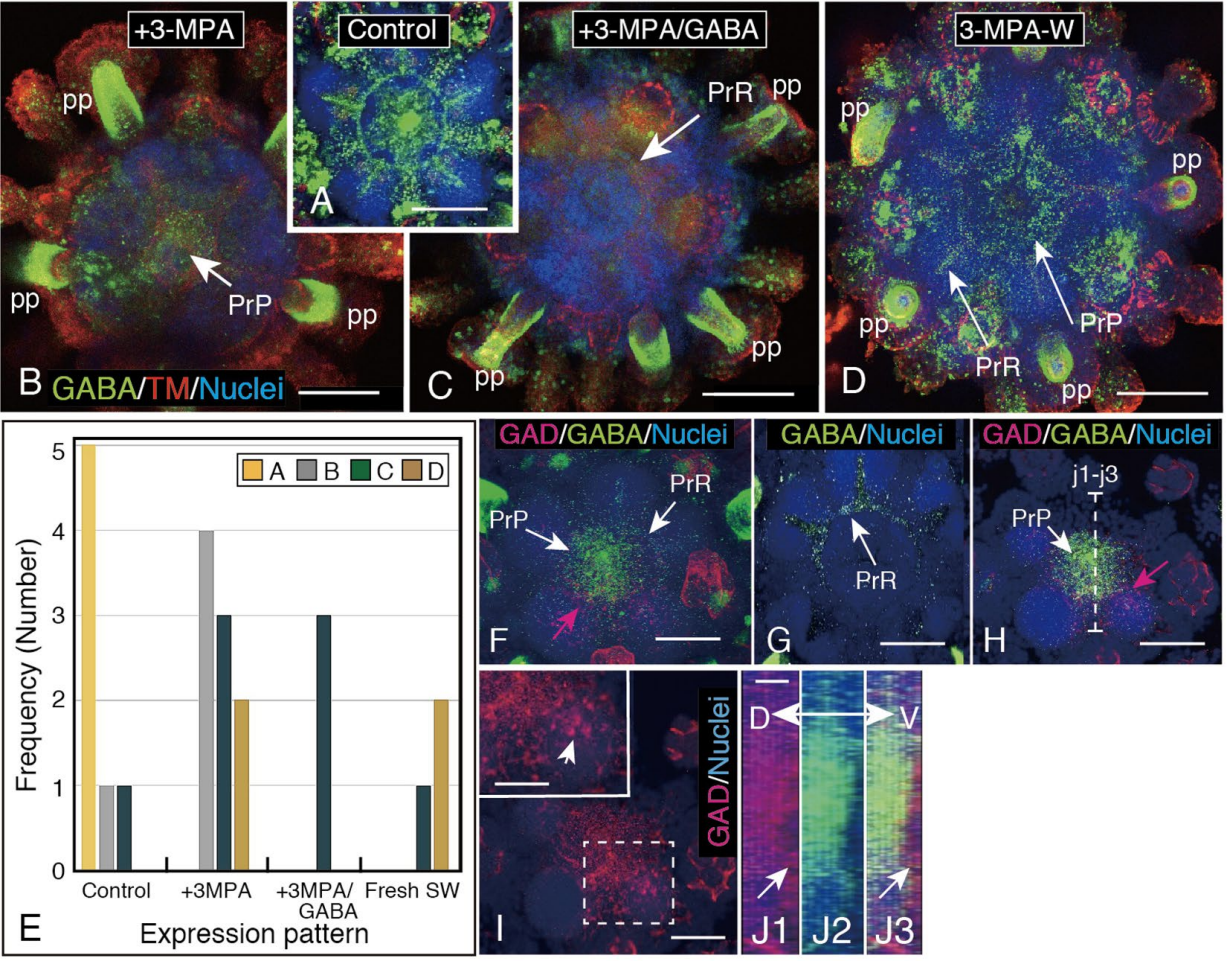
3-MPA-sensitive and-insensitive GABA-IS organs in juveniles. (**A**) GABA-IS Penta-radial nerve ring (PrR)/Penta-radial plate (PrP) complex in Control juvenile. (**B**) GABA-IS is inhibited at the PrR by 3-MPA, while it is not at the gaiter region of all primary podia (pp). (**C**) GABA-IS is weakened or inhibited at the PrR/PrP complex but is not affected at PP by a mixture of 3-MPA and exogenous GABA. (**D**) GABA-IS is restored at the PrR/PrP complex after washing with normal seawater. (**E**) Frequency of GABA-IS expression pattern of juveniles in seawater alone (Control), with 3-MPA (3-MPA), with 3MPA and exogenous GABA (3-MPA/GABA), and in plain seawater after washing out 3-MPA (Fresh SW). GABA-IS patterns A (yellow column), B (gray column), C (deep green column) and D (light brown column) encased in a rectangle are represented respectively by above WMIHC images (**A**–**D**). (**F**) 3-D reconstructed images of PrP. Optical cross-section of the oral side of the PrR/PrP complex by ImageJ. (**G**) Twenty-six μm thick optical cross-section of the aboral side of (**F**) shows GABA-IS PrR alone. (**H**) Fourteen-μm-thick optical cross-section of the oral side of (**G**) shows GABA/GAD-IS PrP. (**I**) GAD-IS plexus after the digital removal of GABA-IS from (**H**). Inset; GAD-IS plexus cells (arrow) by a higher magnification of a box in the mainframe. (**J1**) Sagittal optical cross-section of GAD-IS plexus at the dotted line (j1–j3) in (**H**). (**J2**) GABA-IS plexus of the same area as (**J1**). (**J3**) Merged image between (**J1**) and (**J2**) shows the GAD-IS region at slightly broader area than that of the oral side (Arrow). Double-headed arrow; Dorsoventral axis. Scale bars = 100 μm (**A**–**D**), 50 μm (**F**–**I**), 25 μm (Inset of **I**),10 μm (**J1**).

Above 3-MPA-induced inhibition of the juvenile motility implicated a decline of GABA-IS in the PP. However, the present stacked image of the CLSM detected little decline of GABA-IS at gaiter region of PP. (Fig. 8B). This revealed that GABA-IS in gaiter region of PP is little or not involved in the creeping movement of the juveniles. However, GABA-IS was also detected in the PrR/PrP complex of the juvenile (Figs. 6 and 8A). These regions lost GABA-IS in the presence of 3-MPA (Fig. 8B), and the GABA-IS was scarcely restored even by the addition of exogenous GABA (Fig. 8C, PrR). Washing the juveniles with plain FSW for 1 to 2 h significantly restored the GABA- IS (Fig. 8D), although the immunosignal intensity was not fully recovered to the level that was shown by the control. This could contribute to the incomplete restoration of motility (Fig. 1C). The representative WMIHC images of the resultant of the above four experiments were summarized by a frequency distribution graph (Fig. 8E) that indicated considerable similarity with the above swimming activity (Fig. 1C).

Next, to examine what provided GABA to the PrR/PrP complex in the absence of the exogenous GABA source, the presence of the endogenous GABA supply system, a GAD-IS site, was examined using 3-D image reconstructed CLSM images. The 3-D image detected the GABA-IS PrR/PrP complex being accompanied by the GAD-IS area (Fig. 8F, red arrow). An optical 26 μm thick cross-section that was dissected horizontally between GABA-IS PrR and PrP, which was consistent with above Fig. 7B, had only GABA-IS PrR (Fig. 8G), while the other side of the cross-section contained GAD-IS region (Fig. 8H, red arrow), which was closely associated with GABA-IS PrP (Fig. 8H). The single filter analysis of the oral PrP region detected the GAD-IS cell plexus (Fig. 8I, inset arrow). To further analyze the spatial relationship between the GABA-IS region and the GAD-IS region of PrP, an optical cross-section was cut perpendicularly through the middle of PrP was produced (Fig. 8H, dotted line j1-j3). The optical cross-section showed the GAD-IS region throughout the oral-aboral axis of PrP (Fig. 8J1) and a little thinner GABA-IS region (Fig. 8J2). The merged image between these two revealed a slightly wider GAD-IS region to the oral side of the PrP (Fig. 8J3, arrow). This suggested the occurrence of GAD-IS/GABA-IS area interactions in PrP and, thus, a possible GABA supply from PrP to PrR and then to the proximal region of the PP. This further verified that the GABAergic regulatory system of juvenile motility resides at the PrR/PrP complex but may not be in the PP.

## Discussion

The “basal blastocoel GAD-IS-network” contacts to the ciliary band-associated strand (Fig. 2A,B), which suggests some signal transmission between them^1,20,21^. A part of the network reaches also to the EcR surface and PP. Thus, the body surface GAD-IS cells that contact the distal spiny projections of PP in juvenile may be derived from the GAD-IS network in plutei^22^. Although the biological role of these body surface GAD-IS cells other than the GABA supply needs to be examined in the future, some other molecules could contribute to the 3-MPA resistant GABA supply to the gaiter region of PP, such as aldehyde dehydrogenase1a1 (ALDH1a1)^23^ and the similar molecule is reported in sea urchins, such as ALDH1 in *H*. *pulcherrimus* by HpBase (http://cell-innovation.nig.ac.jp/cgi-bin/Hpul_public/Hpul_annot_search_output.cgi)^24^ and in *S*. *purpuratus* by SpBase (https://urchin.nidcr.nih.gov/cgi-bin/exp.plx). This will be discussed later.

A large abdominal hole detected above EcR and hole-oriented alignment of all five PP may indicate that the hole is an exit for the adult form in the final stages of metamorphosis [PP-touching stage viii^25^], and its formation may include the organ rearrangement mechanism, such as apoptosis^26,27^.

During the final stage of metamorphosis, the 8-arm pluteus larva occasionally extends PP to anchor or creeping on the substrate. This is interrupted frequently by swimming with the ciliary beating at the epaulettes, which implies that their respective motilities are not interrupted by each other. Thus, both neuroregulatory systems are valid, and the larval GABAergic system remains active despite the ongoing absorption of the larval organs, as has been reported for the remained partial larval organs in *H*. *pulcherrimus*^6^. A part of the mechanism of the irrespective movement of these motile organs may be due to disconnection of adult and larval nerve fiber^27^ at the node nearby EcR^7^.

The pharmacological response of TF to GABA in major echinoderm species^12,28,29^, and its immunohistochemical detection was reported in starfish^11^ and sea cucumber^30^, but not in sea urchins to date. The present striking GABA-IS site was the gaiter region of the juvenile PP. Along with the GABA_A_R-IS layer on the muscle layer, this resembles GABA_A_R involvement in the neuromuscular junctions as has been reported in *C*. *elegans*^31^. It also suggests GABA involvement in PP motility-related osmotic regulation via GABA_A_R signaling^32^, such as that reported in GABA-induced depolarization of echinoderm TF muscle preparations^12,28^.

The GAD-IS was seen accompanied by GABA-IS in PP from the later stages of metamorphosis to juvenile. The distal tip of the PP radiated numerous projections toward the GAD-IS network of the body-surface, and they are apparently in contact with each other. This may indicate a morphological GABA transmission pathway to the PP. In larvae, ciliary band-associated strand that contains GAD transmits GABA to CB via synaptophysin-involved mechanism^14^. Thus, synaptophysin mediation could be predicted in GABA transmission to the gaiter region of the PP.

The present study detected 3-MPA-insensitive or low-sensitive GABA-IS at the gaiter region of the PP, which reminds inhibitor-insensitive GAD activity in mice and the fish, *Salmo irideus*^33^ or GAD independent GABA syntheses, such as glial monoamine oxidase B in the cerebellum and the striatum of adult mice^33^. Regarding our previous reports of the GAD expression in ciliary band-associated strand^13,14^ and the dopamine expression at nearby CB^6^, involvement of the GAD-independent ALDH1a1-dependent GABA synthetic pathway in *Xenopus* tadpole and mammalian cells^23^ may also be a possible interpretation. Regarding that ALDH1 and the likes are reported in sea urchin genome databases (HpBase; http://cell-innovation.nig.ac.jp/cgi-bin/Hpul_public/Hpul_annot_search_output.cgi, SpBase; https://urchin.nidcr.nih.gov/cgi-bin/exp.plx?ind=1amp;cname=Sp-Aldh1amp;glean=GLEAN3_01700), such possibility can be considered.

In the PrR/PrP complex, both components are connected via several intra-structural GABA-IS cables (Fig. 7A), which suggests that GABA is synthesized at the GAD-IS cells in/near PrP and transmitted to PrR through the cables, and the neurotransmitter is delivered from there to PP to regulate the creeping movement. Furthermore, the neurotransmitter may first reach the GABA_A_R-IS layer on the outer surface of the muscle layer of PP, which suggests the occurrence of the receptor-mediated signal transmission in the regulation of muscle contraction for the creeping movement. The presence of GABA_A_R on the muscle layer of PP is consistent with an electrophysiological report on the holothurian neuromuscular junction^29^. This suggests the occurrence of the excitatory responses to PP, as has been predicted in echinoderms^30^ and is apparently consistent with the present inhibition of the creeping movement of juveniles by 3-MPA application. This will be further discussed in the next section. A similar GABA-IS radial nerve cord and that in PP were reported in the asteroid *Asterias rubens*^11^. However, such organization as the PrR/PrP complex has never been reported to date in other echinoderms.

The reversible GABA-IS vanishing at the PrR/PrP complex following the administration of 3-MPA, which resulted in the inhibition of creeping motility of juveniles, while that in the gaiter region of PP has remained unaffected. This implicates that there are different effects of the inhibitor on GAD activity between in the PrR/PrP complex that contributes PP activity and the other near the body surface and the gaiter region that is not involved in PP motility. The present inhibitory effect of 3-MPA for juvenile motility implicates that GABA acts as an excitatory neurotransmitter on the PP musculature, as has been widely reported for the echinoderm such as the sea urchin *Strongylocentrotus franciscanus*^12^ and the starfish *Asterias amurensis*^28^. Involvement of GABA_A_R in GABA signal transmission has also been reported to be excitatory in the sea cucumber, *Holothuria glaberrima*^30^. Unlike the GAD-IS plexus of the PrP/PrR complex for the Penta-radial ring, the GABA source for the gaiter region of PP could not solely depend on GAD activity, as has been suggested the GAD-independent GABA synthesis pathway, such as putrescine, as an initial substrate reported in mouse and fish brain^33,34^ and sea urchin^35^. Although >90% of GABA synthesis activity by GAD67 subunit against GAD65 subunit has also been reported^36^, no such heterogenous subunit complex has been reported in the sea urchin genome (HpBase: http://cell-innovation.nig.ac.jp/cgi-bin/Hpul_public/Hpul_annot_search_output.cgi ^24^) to date. Thus, the molecular basis by which to interpret the different responsiveness of GAD in sea urchins needs to be examined in the future. It also would be important to extend this investigation to the definitive juvenile.

## Supplementary information

Supplementary information.

Supplementary information 2.

Supplementary information 3.
